# Patient safety culture in neonatal intensive care units: A qualitative content analysis

**DOI:** 10.3389/fpubh.2023.1065522

**Published:** 2023-01-20

**Authors:** Mohadese Babaie, Manijeh Nourian, Foroozan Atashzadeh-Shoorideh, Houman Manoochehri, Malihe Nasiri

**Affiliations:** ^1^Student Research Committee, Department of Pediatric and Neonatal Intensive Care Nursing, School of Nursing and Midwifery, Shahid Beheshti University of Medical Sciences, Tehran, Iran; ^2^Department of Pediatric and Neonatal Intensive Care Nursing, School of Nursing and Midwifery, Shahid Beheshti University of Medical Sciences, Tehran, Iran; ^3^Department of Psychiatric Nursing and Management, School of Nursing and Midwifery, Shahid Beheshti University of Medical Sciences, Tehran, Iran; ^4^Department of Basic Sciences, School of Nursing and Midwifery, Shahid Beheshti University of Medical Sciences, Tehran, Iran; ^5^Department of Biostatistics, School of Nursing and Midwifery, Shahid Beheshti University of Medical Sciences, Tehran, Iran

**Keywords:** safety culture, neonatal intensive care units, directed qualitative content analysis, nurses, physicians

## Abstract

**Background:**

Safety culture, as an important and influential component of neonatal safety, can lay the ground for the provision of professional and quality care by creating a positive insight among workers. The present study aimed to explain the concept of safety culture and its dimensions from the perspective of the nurses and the physicians working in neonatal intensive care units (NICUs).

**Methods:**

This qualitative directed content analysis study was carried out with 24 NICU physicians and nurses working in Tehran, Iran. These multicenter participants were selected through purposive sampling with maximum diversity in terms of demographic characteristics. The data was collected through in-depth semi-structured interviews and was analyzed using the deductive approach. The COREQ checklist was used for the comprehensive report of this study.

**Results:**

The concept of patient safety culture in NICUs included achieving professional development, constructive interactions, organizational supportive climate, management's commitment to neonatal safety, planning and implementation of neonatal developmental care, which are extracted from 5 main categories, 10 generic categories and 21 sub-categories.

**Conclusion:**

The dimensions of safety culture include procedures that, if promoted, could improve neonatal safety, reducing harm to neonates' health while expending less financial and human resources. Gaining knowledge of the status of these dimensions in wards and hospitals can give a purposeful direction to promote neonate health and policymaking.

## Introduction

Patient safety is the first priority of healthcare systems. However, not enough attention has been paid to it in neonatal intensive care units (NICUs) (1). The NICU is a complex care environment; however, technology has improved premature infant survival and quality of life (2, 3). Premature infants endure invasive lifesaving diagnostic and therapeutic approaches daily (4). Long epochs of separation from parents, lack of sensory-environmental support, and repeated painful procedures are considered traumas that cause exaggerated hypothalamic-pituitary-adrenal axis (HPA) which results in increased cortisol and potentially leading to dysregulation of the HPA axis (5, 6). The fragility and vulnerability of these neonates (7) makes it imperative to create a safety culture.

Implementation of trauma-informed care in the NICU requires that all NICU providers (doctors, nurses, dieticians, physical and occupational therapists) work together to create an environment that is conducive to healing (8). The prerequisite for providing quality care is to maintain neonatal safety, which is effective in preventing medical errors, reducing physical and neurological damage thereby decreasing mortality (9). In order to minimize errors and provide better care, it is important to implement a safety culture (4). Safety culture plays a critical role in achieving a safe organization (10) and improving the quality of care as a suitable concept for measuring patient safety interventions (3, 11).

In fact, delivering appropriate care services demands a positive safety culture among the staff, especially nurses and physicians (12–14). Organizations with a positive safety culture take preventive measures with a common perception of the importance of patient safety (12), and professionals pay more attention to applying safety policies and procedures in a matter of care. This shared vision creates an interprofessional collegial atmosphere between the healthcare staff in high-risk and damaging situations as patient safety becomes the priority. Defining the safety culture is the first step to examining and improving patient safety (13).

Conducting extensive research on the concept of safety culture and the various results obtained suggests the challenging nature of this concept among experts, during the last decade and a half (15). Safety culture is a multidimensional concept, the definition of which is associated with ambiguities (15, 16).

Some define safety culture as a subset of organizational culture that can vary in different departments, specialties, and professional groups (17), in contrast, others regard it as values, attitudes, competencies, and behavioral patterns (18). In some studies, the terms “safety culture” and “safety climate” are deemed synonyms (19). In the most comprehensive definition presented, safety culture consists of issues around the “Overall perceptions of patient safety”, “Frequency of events reported”, “Communication and openness”, “Manager expectations and actions promoting patient safety”, “Organizational learning”, “Teamwork within units”, “Feedback and communication about error”, “Non-punitive response to errors”, “Staffing”, “Management support for patient safety”, “Teamwork across units”, and “Handoffs and transitions” (20). The combination of these dimensions has led to the development of the “Hospital Survey on Patient Safety Culture (HSOPSC)” questionnaire, which is the most accurate tool to assess the safety culture from the staff's perspective (15, 16).

In many NICUs, there is a shortage of information on safety culture (3). The different nature of care in these units, premature neonates' high sensitivity and physical differences, and considering mothers and neonates as a single care unit; make the safety culture in NICUs distinctive from other units, which may have different meanings and dimensions.

Overall, previous research in Iran (14, 21) and other countries (3, 11), often using quantitative approaches and tools tailored to other communities and social structures, failed to reach a universal consensus on the definition of safety culture as a concept, which is directly related to the quality of care (22). Considering the multidimensionality of the concept of safety culture and the specific conditions of neonatal intensive care units, it seems that quantitative studies alone cannot identify this complex concept. In-depth qualitative methods are better suited to accessing more profound aspects of safety culture (23).

## Purpose

Recognizing the safety culture concept as an important factor in maintaining neonatal safety, especially from the perspectives of nurses and physicians who have first-hand experience in this field, plays an important role in hospital policymaking and providing safe care approaches. Therefore, the present study is conducted using a qualitative approach, aiming to explain the concept of safety culture and its dimensions in NICUs and to better and more deeply understand nurses' and physicians' perspectives on this concept.

## Methods

### Study design

This multicenter, qualitative study was conducted to explore the meaning of patient safety culture from the perspective of nurses and physicians working at NICUs in 12 hospitals affiliated to the Universities of Medical Sciences, from late April 2019 to March 2020 in Tehran, Iran.

### Setting

These selected hospitals are the most prominent metropolitan educational and treatment centers for the referral and hospitalization of premature infants in need of intensive care in Iran.

### Participants

Based on purposeful sampling, an appropriate method for qualitative study participant recruitment (24, 25), 35 staff were invited and 24 staff (15 Bachelors/Masters and PhD in Nursing, and nine physicians, including neonatologists, fellows, pediatricians, and pediatric assistants) agreed to participate in the study. The inclusion criteria consisted of physicians and nurses physically and psychologically healthy (according to their reports and medical records), a minimum of 1 year of experience in the NICU, with the ability and willingness to participate in interviews. The maximum diversity in terms of demographic characteristics was considered gender, age, marital status, level of education, work experience in NICU, and shift status. Nursing managers (matron and supervisors), and individuals who were not directly involved in neonatal care were excluded.

### Data collection

Data were collected using one-on-one, in-depth semi-structured interviews based on the structure of the HSOPSC questionnaire, with open-ended questions (Table 1). The HSOPSC is developed by the Agency for Health Care Research and Quality (AHRQ) and explores the concept of safety culture in 12 dimensions (20). The survey of this instrument, examining 1,437 hospital workers, reported acceptable levels of Cronbach's α internal consistency (0.63–0.84) and construct validity (26).

**Table 1:** Interview guide based on HSOPSC questionnaire.

**Dimension of HSOPSC**	**Question**
Overall perceptions of patient safety	What do you think neonatal safety means? What comes to your mind when you hear the word safety? What makes the safety of the neonates?
Frequency of events reported	How did it impact the patient when you or one of your partners made an error in the unit? In such cases, what is the unit's approach to error management?
Communication and openness	Regarding your relationship with your partners, which factors enable you to discuss an error with each other if one occurs?
Feedback and communication about error	How will you be notified if one of your partners makes an error? What happened after you reported an error to you or one of your partners'?
Management support for patient safety	How does the hospital's president or the unit's manager contribute to neonatal safety? What is the impact?
Non-punitive response to errors	Based on your experience, have you or your partners ever worried about the consequences of reporting a mistake you made? Can you give an example?
Staffing	How do you think issues such as high workload or the shortage of staff in the unit impact neonatal safety?
Teamwork within units	How does teamwork impact neonatal safety in your unit?
Teamwork across units	How do you think the relationship between your unit and other wards of the hospital impacts neonatal safety?
Organizational learning	How does your unit or the hospital help you learn from the errors made by you or your partners?
Handoffs and transitions	Has neonatal safety ever been compromised during shift change, neonatal transfer to another ward, or at the time of admission? Can you tell me more about it?
Manager expectations and actions promoting patient safety	How do you think the activities of managers, such as the hospital's president, matron, head nurse, or the head of the ward affect neonatal safety? How do managers' expectations of you impact neonatal safety?

In addition, questions such as “What do you mean” or “Could you explain it more clearly”, taking notes during and after the interview, and the careful observation of nonverbal messages and behavior helped achieve a deeper understanding of the concept of safety culture.

The first author, who received thorough training in qualitative studies and has experience in teaching and working in the safety field, before the research, offered some information on the research objectives and the approximate duration of the interview. Written informed consent and permission to audio record the interviews were obtained from the participants before the interview commenced. The researcher also ensured that participants were aware of the confidentiality of the information and the right to withdraw at any time. Participants agreed on the location (rest room in the hospital, office, college, or park) and the time of the interviews, which were conducted in a quiet environment.

The interviews were conducted face-to-face and in the absence of other people. In qualitative studies, sampling continues until data saturation (27). In this study, although data saturation was achieved after interviewing 20 participants, four more interviews were conducted to further ensure data saturation. The interviews took from 25 to 55 min.

### Approach

The directed (deductive) qualitative content analysis (DQCA) method was performed during participant interviews. This method is used when there are incomplete findings of previous research about a phenomenon, and further research is necessary to clearly understand and explain that phenomenon (28). Whereas several studies on patient safety culture have been done, specifically based on the definition and structure of the HSOPSC questionnaire (12, 29), this method was applied to the analysis. Also, the Consolidated criteria for Reporting Qualitative research (COREQ) checklist (30) was used for the comprehensive report of this study.

### Data analysis

The DQCA method was conducted using the Elo and Kyngäs' approach in three phases (31). In the first phase (preparation), each interview was recorded and transcribed, each text was reviewed several times to immerse the data. Then, in the second phase (organizing), the researchers developed a formative categorization matrix to place the codes into predetermined categories for analysis. During data analysis, using MAXQDA 10, the entire text of each interview was considered as an analysis unit. The expressions extracted from the participants' statements regarding the various aspects of the concept were identified as meaning units. Then the primary codes were obtained by the integration of the meaning units and were extracted and classified based on their similarities with the matrix. Coding was also done to other meaning units that were not related to the main categories but were related to the concept of safety culture. This allowed the emergence of new main categories. The codes were placed in the main categories, generic categories, and subcategories were formed through comparison with the categories of the matrix, using the constant comparison method. The matrix was gradually modified and finalized in a way that the obtained themes explained the concept of safety culture in NICUs which is reported in the third phase (reporting).

### Trustworthiness

Speziale et al.'s criteria include credibility, transferability, dependability and confirmability, were considered (25). In order to peer-check the process, the interviews were reviewed by the research team after being codified. External reviews were done by a faculty member and a PhD student other than the research team members. Participant reviews were also carried out by two physicians and one nurse, who were randomly selected from among the participants, in order to confirm the results' accuracy. Purposeful sampling with maximum diversity contributed to data transferability. In addition to a complete report of all the phases that had been gone through and the descriptions of the analysis process, the participants' quotations were recorded to prove that the findings originate from the data.

### Ethical considerations

This article is a part of the PhD dissertation in nursing, approved by the Ethics Committee of Shahid Beheshti University of Medical Sciences under the ethics code IR.SBMU.PHARMACY.REC.1397.270. All participants were informed about the confidentiality of the data and written informed consent was obtained. The location and the time of the interviews were agreed upon by the participants. The researcher also obtained permission to audio record.

## Results

Twenty-four physicians and nurses were interviewed. The demographic characteristics can be seen in Table 2. In the analysis of the interviews, 1,216 primary codes were obtained. After merging similar codes, 75 codes with a frequency of 793 remained. At the end of the categories, a total of 5 main categories, 10 generic categories, and 21 subcategories were extracted (Table 3), which explains the safety culture.

**Table 2:** Participations demographic characteristics (*n* = 24).

**Characteristics**		**Number (%)**
Sex	Female	18 (75)
	Male	6 (25)
Marital status	Single	9 (37.50)
	Married	14 (58.33)
	Divorced	1 (4.17)
Level of education	Bachelors in Nursing	5 (20.83)
	MSc student in nursing/ Masters in Nursing	6 (25)
	PhD candidate in nursing	2 (8.34)
	Pediatrician/ Pediatric resident	6 (25)
	Neonatologist/ Neonatology fellowship	5 (20.83)
Age (year)	24–34	7 (29.17)
	34–44	11 (45.83)
	44–54	6 (25)
Work experience in NICU	<5	5 (20.83)
	5–10	16 (66.67)
	>10	3 (12.50)
Shift status	Fixed	10 (41.67)
	Rotating	14 (58.33)

**Table 3:** Main categories, generic categories, and subcategories of safety culture in NICUs.

**Main categories**	**Generic categories**	**Subcategories**
^*^ Achieving professional development	^*^ Acquiring professional competence	^*^ Mastery of clinical skills ^*^ Critical thinking ^*^ The ability of emotion regulation
	^*^ Professional concern	^*^Adherence to job commitments ^*^ Responsibility
Constructive interactions	Interaction and empathy	Staff's cooperation ^*^ Striving for mutual empowerment
	Participatory care	Teamwork in providing care ^*^ Constructive criticism in teamwork
Organizational supportive climate	Innovative climate and efficient supervision	Organizational innovative and creative climate Efficient supervision procedures
	Organizational leading and learning	Employees' in-service training ^*^ Inexperienced staff' leading
Management's commitment to maintaining neonatal safety	Effective management of resources	Manpower protection ^*^ Provision of care assistance equipment and facilities
	A comprehensive and systemic view of error	Fault management based on cause analysis Giving feedback and preventing errors
^*^ Planning and implementation of neonatal developmental care	^*^ Preparing a developmental care environment	^*^ Designing a standard and appropriate space for care ^*^ Attention to the neonatal individualized developmental needs
	^*^ Parental involvement in care	^*^ Parents and neonate as a single unit of care ^*^ Mother as an independent caregiver

^*^All items (main categories and generic/subcategories) were obtained from interviews. The “non-asterisk” components are common with the HSOPSC (selected as framework) or modified. The “asterisk” components are new and obtained based on interviews only.

### Main category 1: Achieving professional development

The concept of safety culture in NICU is rooted in achieving professional development, which includes “acquiring professional competence” and “professional concern”. Mastery of clinical skills, critical thinking, and management of nurses' and physicians' emotions are considered important factors in health care.

“*The professional competence of NICU staff is important. We had a specialist partner who was intensely stressed in critical conditions with severe impact on her performance. During this working shift, we had IVF twins with sensitive conditions. Our partner was so anxious that she could not make a timely decision and carry out the right procedure. Unfortunately, both neonates passed away…”* (Physician 3).

On the other hand, caregivers must adhere to job commitments and ethical requirements and act responsibly and responsibly in the serious matter of caring for the infant.

“*Safety culture is a reminder that everyone should be committed to professional and moral obligations, and make sure not to harm the neonate as a result of negligence and carelessness...”* (Nurse 10).

### Main category 2: Constructive interactions

Another dimension of the concept of safety culture in the NICU is constructive and desired professional interactions, which include “interaction and empathy” and “participatory care”. The staff's cooperation with each other in providing care and support is an example of desirable interactions and empathy in the unit. Moreover, through professional interactions, partners have an opportunity to benefit from each other's skills and expertise.

“*The NICU staff should be different from other staff, the way they cooperate, the support the offer each other, or the way they work to improve each other's performance. This kind of interaction is valuable...”* (Physician 7).

Adherence to the values and the principles of teamwork in providing participatory care and creating an environment where the staff can freely express their opinions and criticisms of infant safety issues shows a clear picture of safety culture.

“*At the NICU, we literally see team care and effective professional communication among physician and nurse partners. Anyone entering the NICU should have the attitude to criticize the status quo as a team member, and to notify anyone who has forgotten something and, of course, the other person must accept it too…”* (Nurse 15).

### Main category 3: Organizational supportive climate

An organizational supportive climate is defined as an “Innovative climate and efficient supervision” and “organizational empowerment and learning”. The concept of safety culture is realized in an organizational environment that is a creative environment and encourages employees to come up with innovative strategies to improve neonatal safety. It all depends on supervisory procedures contribute to the implementation of this culture.

“*If the organization's strategy is to value these creativities, everyone is encouraged to come up with ideas and there would no longer be any need to give the staff a scale”* (Nurse 1). “*One of the most valuable things is the work done by the Neonatal Health Department of the Ministry of Health, which, based on field studies, is planning to more accurately follow up the issued topics. I don't want to imply that by doing these, we have met the NIDCAP*^1^ *standards, but we have long passed the disorganized health care…”* (Physician 4).

The interviewees emphasized the need for in-service training, especially for the new staff, as a major factor in changing behaviors, improving the effectiveness of neonatal care, and achieving organizational learning that leads to a mental transformation in the staff and forms a common goal.

“*In-service training is an important process in any organization with specific funding. In the health system, where we are dealing with people, the issue becomes bolder, especially in regard with premature neonates, and should be specifically addressed...”* (Physician 8).

### Main category 4: Management's commitment to maintaining neonatal safety

Management's commitment to neonatal safety includes “effective management of resources” and “a comprehensive and systemic view of error”. Addressing the important issue of manpower protection with the aim of increasing occupational motivation and satisfaction and the provision of safe care assistance equipment requires principled managerial actions to provide and allocate funding to these resources, and determine the appropriate procedure for related follow-ups.

“*It is true that the head of the unit manager supports us, but we did not receive the support we needed from the hospital management. Such attention creates a sense of security and satisfaction…”* (Nurse 6). “*When a device or apparatus is in short supply, or needs to be repaired, medical equipment providers and maintenance department should properly cooperate. In these cases, managers have to make rules to facilitate these types of access...”* (Physician 2).

While encountering an error, it is necessary for the management to adopt a systematic approach, with the aim of examining the personal and systemic reasons for the error from a different and holistic perspective, instead of punishing the one making the error, and sharing the results with the staff and give feedback on the required corrective actions to the employees.

“*The right thing to do is to forget the old way of reprimanding the wrongdoer, and to fundamentally examine the whole system, in order to identify and eliminate the real causes…”* (Nurse 13). “*The best outcome is obtained from raising the staff's awareness. As long as the management does not inform the staff of these incidents and solutions, we will experience tragic events...”* (Physician 9).

### Main category 5: Planning and implementation of neonatal developmental care

The last dimension of safety culture in the NICU is proposed as “preparing a developmental care environment” and “parental involvement in care”. Designing a caring environment for developmental support, concurrently with providing medical care, is of great importance and an indicator of the safety culture in the unit. All healthcare methods such as position change, pain management, and supporting sleep-wake cycles should be planned in a way that facilitates neurodevelopment.

“*Most of the infant's neurodevelopment happens in the ward. It makes us so sensitive to the care. We turned down the sound of alarms and turned off extra lights especially during evening and night shifts. If a surgery is done, we relieve the pain, and we really try to give them a good rest. We also have KMC in the care...”* (Nurse 3).

According to the safety culture, the turning point of care is the family and the neonate. In all safety considerations, parents, especially the mother and the neonate, are considered as a unit of care. In addition, a part of developmental care focuses on maternal empowerment as a therapist.

“*The neonate is not separate from its parents. It is the provision of specialized care for the neonate and the family. So, there would be mental and even physical harm to the parents, especially the mother, which should not be ignored. Moreover, the mother plays a vital role in accelerating recovery...”* (Nurse 8).

## Discussion

Safety culture can lay the ground for neonatal safety by making a systematic change in the staff and managers' perspectives. The findings of this qualitative study, based on the structure of the HSOPSC, led to the extraction of more detailed context-based information about the safety culture. Providing the characteristics of each dimension explains this concept from the NICU nurses and physicians' perspectives. The reports obtained from these demographically diverse samples supply valuable insight into this concept.

The concept of safety culture in NICUs was similar to the structure of HSOPSC; however, in some categories and details, it is very distinctive. The participants referred to the necessity of safety culture in neonatal care and its implementation in the unit and hospitals as a basic framework for safe practice and attitude. They regard safety culture as an organizational culture that prioritizes safe neonatal care. Managers and all staff take responsibility for its promotion through interaction and empathy. The results of this study are consistent with many other studies. In various studies on the beliefs, values, and attitudes of an organization's employees, individual and group behavioral patterns have been mentioned as the underpinnings of safety culture, which determines an individual's obligations and performance in a health organization (15, 16).

In the present study, the main dimension of neonatal safety culture is the achievement of professional development. Because staff are the main pillars of care provision, it is very important for them to acquire professional competencies, including high knowledge and care skills (10, 32). Many studies consider healthcare professionals as a key factor in safety culture (3, 12, 15, 33). Because responsible and professionally competent staff instill a sense of security and consider the patient's sensitive condition, their specialized skills can be used for the benefit of the neonates' health. This main category and its sub-categories were extracted based on the participants' opinions in the present study, which differs from HSOPSC. Therefore, it is regarded essential dimension of safety culture in NICUs.

Interviewees mentioned desirable and constructive interactions among partners, full of respect and mutual trust. The “Teamwork within/ across units” and “Handoffs and transitions” dimensions of HSOPCS consist of the items which explore how colleagues communicate. First impressions seem to suggest a similarity in dimensions, but the nature and quality of this interaction (“Striving for mutual empowerment” and “Constructive criticism in teamwork”) are distinctive features of the safety culture in NICUs where employees have been trained. Physicians and nurse partners in the unit empathize with each other. This dimension is the most common form of the concept of safety culture perceived by health system workers (12, 17, 29); as approved by previous qualitative studies (10, 17).

In the study by Wami et al., the interviewees believed that conflicts among the staff lead to poor teamwork and negatively affect patient safety culture (29). This issue is especially troublesome during patient handover, changing shifts, or transmitting information (23). On the contrary, coordinated teamwork can lead to appropriate multidisciplinary care (3, 12). A desirable interaction paves the way for the provision of specialty care in a participatory manner. On the other hand, it provides the climate for constructive criticism and its acceptance, regardless of administrative hierarchy and seniority, which leads to promoted neonatal safety. This climate is welcomed by all, and all this is realized within an appropriate context of organizational culture in the hospital.

In fact, another dimension of the concept of safety culture proposed by the interviewees is the depth of support provided by the organization, which is a combination of the “Manager expectations and actions promoting patient safety”, “Management support for patient safety”, and “Organizational learning” from the model. It focuses on development of the rules that facilitate neonatal safety and monitor its implementation. Maintaining neonatal health is the preservation of valuable human resources for the future. Therefore, optimizing the organizational culture by focusing on development of an appropriate vision and strategic planning to strengthen the patient's safety culture (12) is inevitable. Organizational learning was also mentioned as a formal procedure and a vital strategy for the promotion of the staff's knowledge, which is in line with various studies in this field (10, 12, 34, 35).

Management interventions play a key role in improving patient safety (32, 36) and should be given more attention in order to achieve effective communication and the efficient teamwork and obtain positive feedback. Protecting manpower is the most important action that results in increased job satisfaction, organizational belonging, and better performance among caregivers. Allocating funding resources for equipping the unit, providing care equipment, and creating a safe environment are other managerial actions considered by the interviewees, which is in line with previous studies (12, 37). In a qualitative study, the participants referred to an association between staff departure and the decline in the quality of care. They believed that management focuses on budgetary and economic goals instead of paying attention to employees and keeping them; staff and their demands are missed, and the existing problems (lack of equipment and supplies) are considered unimportant (23).

In this study, the importance of managers' supervision on the implementation of safety procedures, having an accident reporting system and forming a risk management and safety committee was discussed (10, 12, 23). But the effectiveness of such a system is questionable because, in general, serious incidents are reported, and there is still a reluctance to report these errors due to the fear of punitive actions by management, feelings of shame, and the loss of partners' trust. Although it is related to the “non-punitive response to errors”, “frequency of events reported”, “feedback and communication about error”, and “staffing” of HSOPSC; however, the provision of care assistance equipment and facilities is the distinguishing feature of this safety culture dimension in the NICU department.

In addition, the planning and the implementation of neonatal developmental care was extracted as the last dimension of the safety culture concept, which differs from the results of previous studies on safety culture. Due to the nature of the developmental care program, family involvement is more highlighted (38, 39).

The role of parental involvement and kangaroo care in the Newborn Individualized Developmental Care and Assessment Program (NIDCAP) (38) in maintaining neonatal neurodevelopment, is apparent to everyone. Kangaroo care is the best trauma-informed care intervention to promote parent-infant bonding through increased oxytocin levels in both mom and infant and relieve the stress. Furthermore, understanding the parent-infant safety-seeking behaviors will help providers use trauma-informed interventions containing respectful, nonjudgmental personalized care (6). The proper implementation of these requires staff knowledge and a positive understanding of and attitudes toward NIDCAP. According to the interviewees, the Neonatal Health Department at the Ministry of Health has taken helpful measures to appreciate and implement this program. Issuing instructions, facilitating mothers' continued presence and recruiting NICU nurses with master's degrees are some of the measures. However, there are still barriers such as high costs, time-consuming implementation of the program, and most importantly, family coordination with the care team.

## Conclusion

The dimensions of safety culture include procedures that, if promoted, could improve neonatal safety, and shortcomings reducing harm to neonates' health while expending less financial and human resources. Gaining knowledge of the status of these dimensions in wards and hospitals can give a purposeful direction to health policymaking, and validly guarantee the health of neonates, as valuable human assets. It is also necessary to hold training courses on this concept for the staff, especially managers.

## Limitation and strengths of the study

The interviewees were selected from the equipped and advanced NICUs in the city of Tehran. Due to the limitations and the shortages in some hospitals in other cities in the provision of equipment, funding, and qualified staff, perhaps conducting a similar study on those units yields a different definition of safety culture. This is what makes the generalization of findings a little difficult. One of the limitations of this study was some staff's lack of cooperation to participate in the interview (Especially physicians) due to dissatisfaction with the system and working conditions. Of those invited for interviews from 12 hospitals, only nine physicians participated in the study. Despite these limitations, it is emphasized that the knowledge obtained from this study can be valuable for neonatal health promotion and clinical applications. To develop this concept, it is suggested that future studies be conducted in other therapeutic settings.

## Data availability statement

The original contributions presented in the study are included in the article/Supplementary material, further inquiries can be directed to the corresponding author.

## Ethics statement

The studies involving human participants were reviewed and approved by the Ethics Committee of Shahid Beheshti University of Medical Sciences under the ethics code IR.SBMU.PHARMACY.REC.1397.270. The patients/participants provided their written informed consent to participate in this study.

## Author contributions

MB and MNo designed the study, reviewed the study materials, and analyzed the data. MNo prepared the ethics submission. MB conducted the interviews. FA-S, HM, and MNa oversaw all aspects of the study's implementation. All authors read and revised the draft and approved the final manuscript.

## Footnotes

^1^The Newborn Individualized Developmental Care and Assessment Program (NIDCAP) aims to prevent the iatrogenic sequels of intensive care and to maintain the intimate connection between parent and infant. It avoids over-stimulation, pain, stress, and isolation while it supports competence, self-regulation, and orientation.
